# Supplementary material for: Protective Effects of Dipterocarpus tuberculatus in Blue Light-Induced Macular Degeneration in A2E-Laden ARPE19 Cells and Retina of Balb/c Mice
Source: Antioxidants (Basel). 2023 Jan 31;12(2):329. doi: 10.3390/antiox12020329 (PMC9952417; doi:10.3390/antiox12020329)

Table S1. List of antibodies for western blot analyses

Name	Catalog	Company, Location, Nation
Anti-Bax antibody	2772s	Cell Signaling, Danvers, MA, USA
Anti-Bcl-2 antibody	PAS-20068	Thermo Fisher Scientific, MA, USA
Anti-Cas-3 antibody	9662s	Cell Signaling, Danvers, MA, USA
Anti-Nrf2 antibody	Ab137550	Abcam, Cambridge, UK
Anti-SOD antibody	Ab13498	Abcam, Cambridge, UK
Anti-MMP2 antibody	40994s	Cell Signaling, Danvers, MA, USA
Anti-MMP9 antibody	2270s	Cell Signaling, Danvers, MA, USA
Anti-iNOS antibody	13120s	Cell Signaling, Danvers, MA, USA
Anti-COX-2 antibody	12282s	Cell Signaling, Danvers, MA, USA
Anti-ASC antibody	67814s	Cell Signaling, Danvers, MA, USA
Anti-Cas-1 antibody	24232s	Cell Signaling, Danvers, MA, USA
Anti-Cleaved Cas-1 antibody	24232s	Cell Signaling, Danvers, MA, USA
Anti-NLRP3 antibody	15101s	Cell Signaling, Danvers, MA, USA
Anti-β-actin antibody	4967s	Cell Signaling, Danvers, MA, USA

Table S2. Primer sequences for RT-PCR analysis

Primer name	Accession number	Sequence (from 5' to 3')	Product size (bp)
TNF-α	AY423855.1		
Forward		CCTGT AGCCC ACGTC GTAGC	70
Reverse		TTGAC CTCAG CGCTG ACTTG	
IL-6	NM_012589.2		
Forward		TTGGG ACTGA TGTTG TTGAC A	200
Reverse		TCATC GCTGT TGATA CAATC AGA	
IL-1β	NM_008361.4		
Forward		CTACA GGCTC CGAGA TGAAC AAC	79
Reverse		CTACA GGCTC CGAGA TGAAC AAC	
NF-κB	NM_001382627.1		
Forward		GTGAGGTCACTCTAACGTATGCAACAGG	361
Reverse		CTCCACCACATCTTCCTGCTTAGTG	
β-actin	NM_007393.5		349
Forward		TGG AAT CCT GTG GCA TCC ATG AAA C	
Reverse		TAA AAC GCA GCT CAG TAA CAG TCC G	

Table S3. Antibodies list for Western blot analyses

Name	Cat. No.	Company
Anti-HSL	4107s	Cell Signaling, Danvers, MA, USA
Anti-p-HSL	PA5-64494	Thermo Fisher Scientific, MA, USA
Anti-ACADs	PA5-54580	Thermo Fisher Scientific, MA, USA
Anti-ACO1	PA5-41753	Thermo Fisher Scientific, MA, USA
Anti-ATP Citrate Lyase	#4332s	Cell Signaling, Danvers, MA, USA
Anti-p-ATP Citrate Lyase	#4331s	Cell Signaling, Danvers, MA, USA
Anti-PPARα	Ab215270	Abcam, Cambridge, UK
Anti-p-PPARα	PA1-820	Thermo Fisher Scientific, MA, USA
Anti-βactin	87809	Cell Signaling, Danvers, MA, USA

Supplement Figure S1. LC-MS analysis of MED and A2E. (A) Seven active components including gallic acid, bergenin, ellagic acid, ε-viniferin, asiatic acid, oleanolic acid, and 2α-hydroxyursolic acid were detected as each different peak in chromatogram.(B) Purity of A2E was confirmed using the gradient HPLC method with a YMC-Triart C18 column. Abbreviations: UV, ultraviolet; A2E, N-retinylidene-N-retinylethanolamin.

A

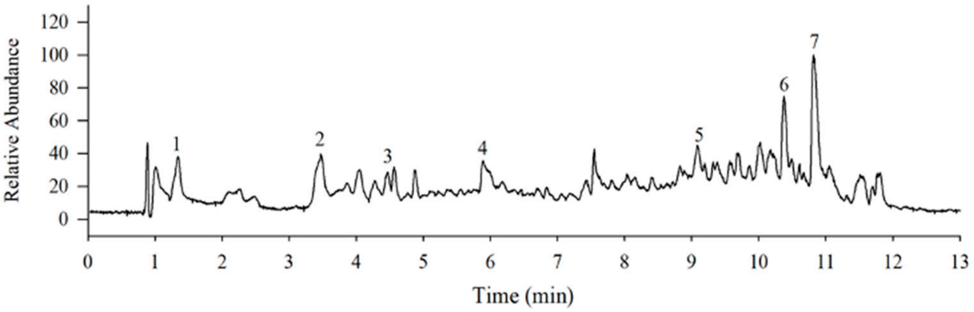

Peak	Compound	Retention Time (min)	Precursor Ion (*m/z*)	Area %
1	Gallic acid	1.34	169.0 [M-H]⁻	2.66
2	Bergenin	3.47	327.0 [M-H]⁻	3.43
3	Ellagic acid	4.46	300.9 [M-H]⁻	1.39
4	ε-viniferin	5.87	453.1 [M-H]⁻	3.21
5	Asiatic acid	9.08	487.3 [M-H]⁻	15.23
6	Oleanolic acid	10.37	455.3 [M-H]⁻	4.11
7	2α-hydroxyursolic acid	10.85	471.3 [M-H]⁻	7.51

B

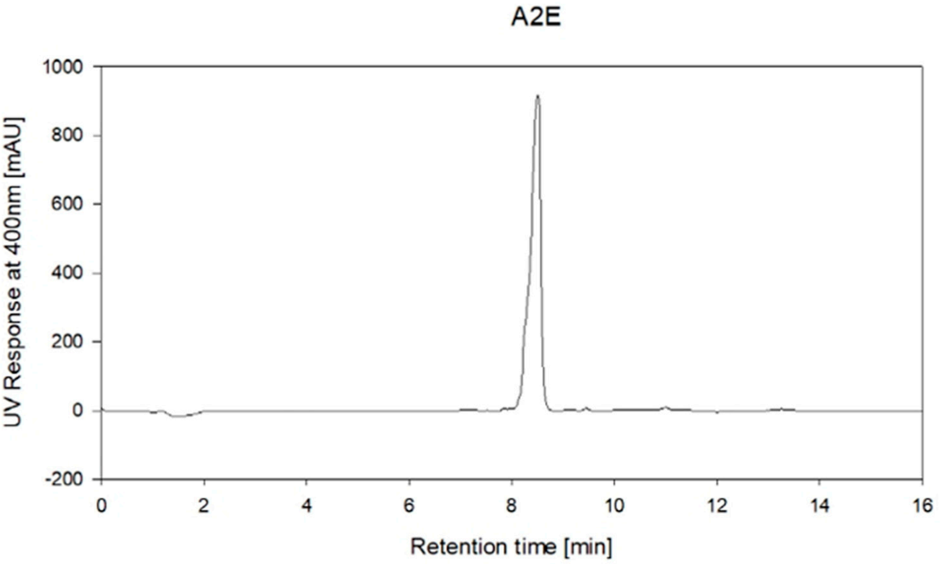

Supplement Figure S2. Determination of optimal concentration of MED. (A) After treating various dose (50, 100, 200 and 400 μM) of MED for 24 h, the viability of ARPE19 cells were detected using MTT assay. (B) These cells were observed using a inverted microscope at 200× magnification. Three to five wells per group were used for MTT assay, and the optical density were repeated for three times. Data represent the mean ± SD of triplicates. *, $p < 0.05$ relative to the Non treated group. Abbreviations: MED, methanol extracts of *D. tuberculatus*.

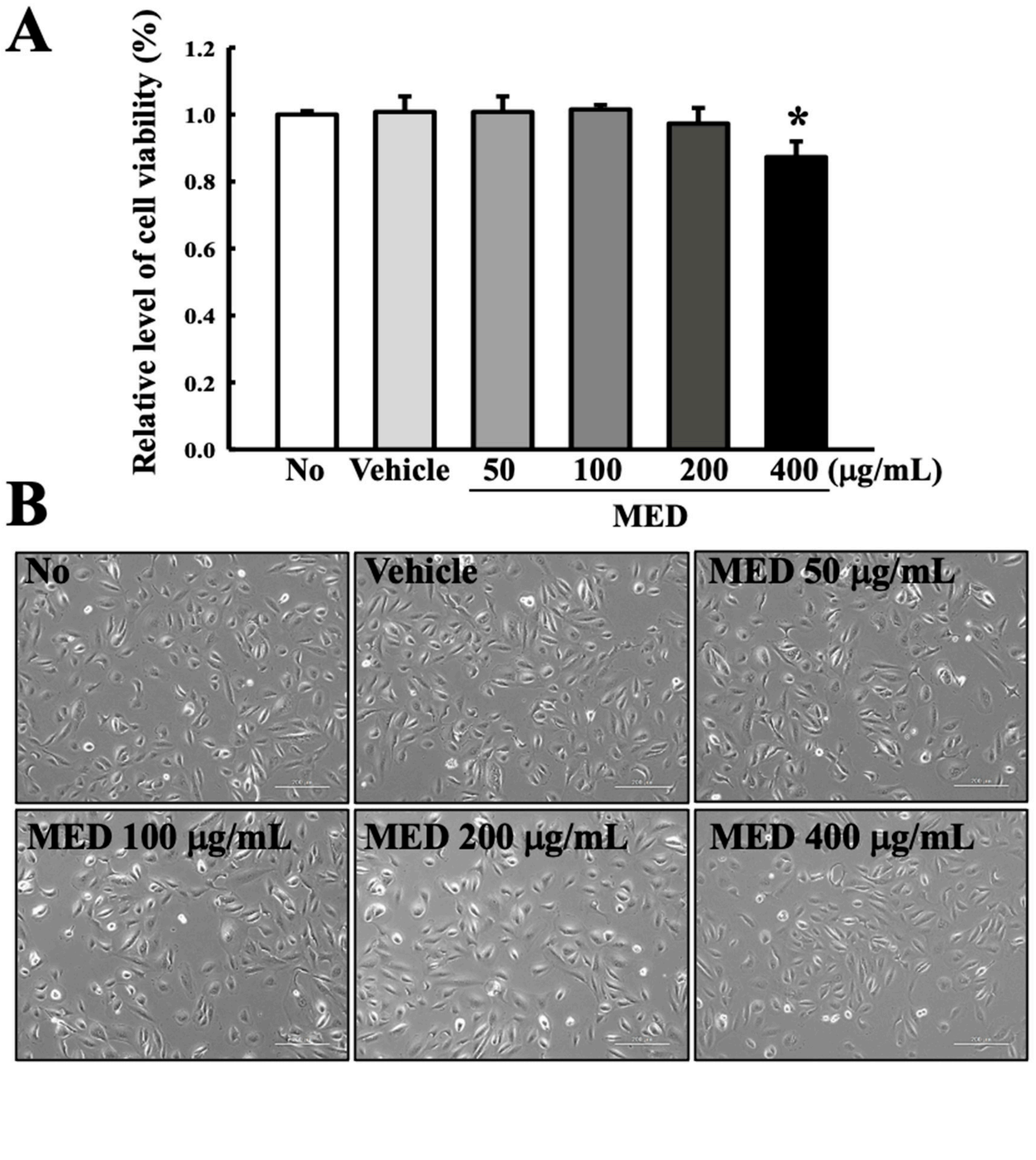

Supplement Figure S3. Determination of optimal concentration of A2E and BL. (A) After treating various concentrations of A2E (5, 10, 20, 40, and 80 μM) for 24 h and irradiated with BL (450 nm, 2,000 lux), the viability of ARPE19 cells were detected using MTT assay. (B) These cells were observed using a inverted microscope at 200× magnification. Three to five wells per group were used for MTT assay, and the optical density were repeated for three times. Data represent the mean ± SD of triplicates. *, $p < 0.05$ relative to the Non treated group. Abbreviations: A2E: N-retinylidene-N-retinylethanolamine; BL, Blue light.

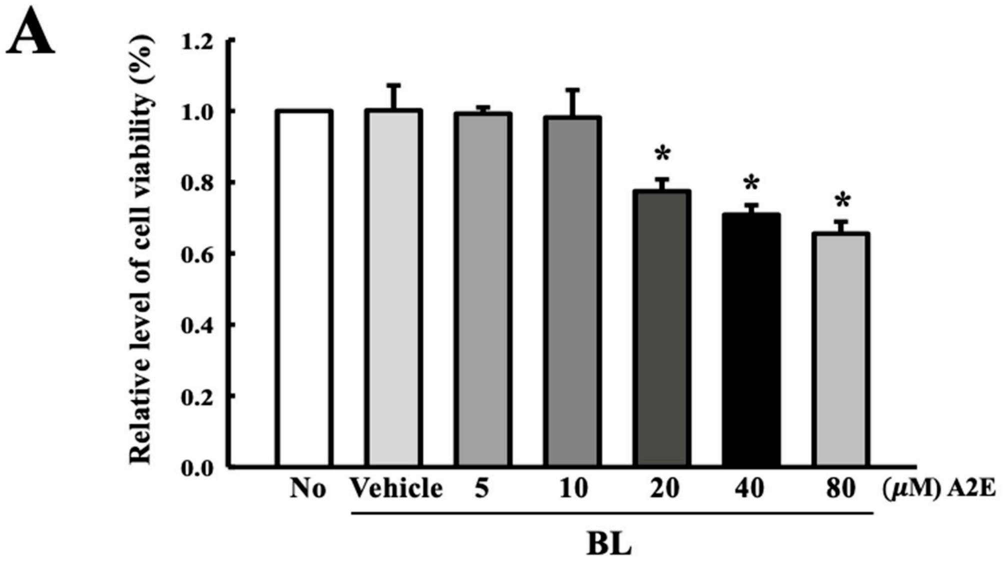

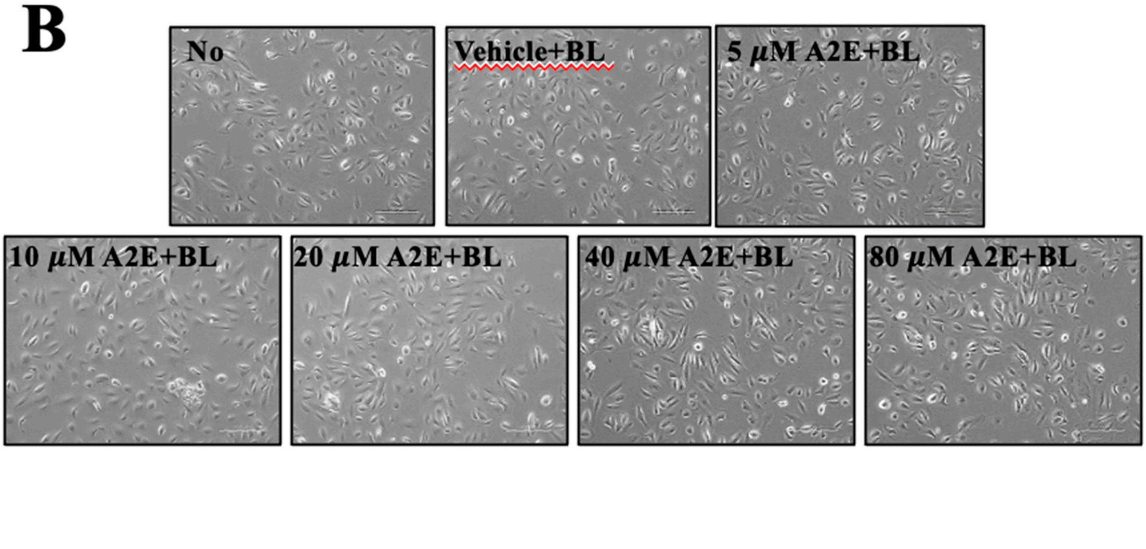

Supplement: Supplementary file 1 [file antioxidants-12-00329-s001.zip › antioxidants-2124091-supplementary.pdf]
